# Hepatitis C Virus E1E2 Structure, Diversity, and Implications for Vaccine Development

**DOI:** 10.3390/v16050803

**Published:** 2024-05-18

**Authors:** Brian G. Pierce, Nathaniel Felbinger, Matthew Metcalf, Eric A. Toth, Gilad Ofek, Thomas R. Fuerst

**Affiliations:** 1 Institute for Bioscience and Biotechnology Research, University of Maryland, Rockville, MD 20850, USA; pierce@umd.edu (B.G.P.); nfelbing@umd.edu (N.F.); mmetcal1@umd.edu (M.M.); eatoth@umd.edu (E.A.T.); gofek@umd.edu (G.O.); 2 Department of Cell Biology and Molecular Genetics, University of Maryland, College Park, MD 20742, USA

**Keywords:** HCV E1E2, structure, nanoparticles, vaccine

## Abstract

Hepatitis C virus (HCV) is a major medical health burden and the leading cause of chronic liver disease and cancer worldwide. More than 58 million people are chronically infected with HCV, with 1.5 million new infections occurring each year. An effective HCV vaccine is a major public health and medical need as recognized by the World Health Organization. However, due to the high variability of the virus and its ability to escape the immune response, HCV rapidly accumulates mutations, making vaccine development a formidable challenge. An effective vaccine must elicit broadly neutralizing antibodies (bnAbs) in a consistent fashion. After decades of studies from basic research through clinical development, the antigen of choice is considered the E1E2 envelope glycoprotein due to conserved, broadly neutralizing antigenic domains located in the constituent subunits of E1, E2, and the E1E2 heterodimeric complex itself. The challenge has been elicitation of robust humoral and cellular responses leading to broad virus neutralization due to the relatively low immunogenicity of this antigen. In view of this challenge, structure-based vaccine design approaches to stabilize key antigenic domains have been hampered due to the lack of E1E2 atomic-level resolution structures to guide them. Another challenge has been the development of a delivery platform in which a multivalent form of the antigen can be presented in order to elicit a more robust anti-HCV immune response. Recent nanoparticle vaccines are gaining prominence in the field due to their ability to facilitate a controlled multivalent presentation and trafficking to lymph nodes, where they can interact with both the cellular and humoral components of the immune system. This review focuses on recent advances in understanding the E1E2 heterodimeric structure to facilitate a rational design approach and the potential for development of a multivalent nanoparticle-based HCV E1E2 vaccine. Both aspects are considered important in the development of an effective HCV vaccine that can effectively address viral diversity and escape.

## 1. Introduction

An estimated 58 million people are infected with hepatitis C virus (HCV) worldwide with 1.5 million new infections occurring each year [1]. HCV infection is a major medical and public health burden in which approximately 75% of infections become chronic, which can lead to liver cirrhosis or hepatocellular carcinoma, and it is a leading cause of liver-related deaths. The number of deaths associated with HCV infection in the United States has been increasing, and it is the primary indication for liver transplantation in the Western world [2,3,4]. The development of direct-acting antivirals (DAAs) has led to cure rates over 90% with HCV treatment, but they do not prevent reinfection. Moreover, DAA treatment is inaccessible to those infected in developing and in developed countries due to high costs and/or infrastructure limitations [5,6,7]. Additionally, the diagnosis of HCV infection often occurs at a late stage after infection due to the asymptomatic “silent nature” of the virus, and successful DAA treatment may not alter the risk for cancer. Reinfection is a particular problem after successful treatment in subjects with continued at-risk behavior such as injection drug use. For these reasons, the most viable method for controlling HCV infections worldwide is through the development of an effective prophylactic vaccine [8].

The structural characterization of the E2 glycoprotein has provided substantial information on the major antigenic sites that are the targets of bnAb binding, particularly as it pertains to binding to primary CD81 receptor binding domain [9,10,11,12,13,14,15,16,17]. More recently, the cryo-EM structural characterization of the E1E2 heterodimer, either as a membrane-extracted E1E2 heterodimer [18] or as a soluble, secreted E1E2 heterodimer ectodomain [19], was a major advance in the field to better understand the structural antigenic features of this complex molecule. This knowledge will greatly facilitate a structure-based design approach in order to optimize immune responses to the major bnAb antigenic determinants. However, the development of a vaccine will be challenging for multiple reasons [20,21,22,23]. These include the genetic diversity of HCV of at least seven HCV genotypes that differ up to 30% in nucleotide sequence (which can be further subdivided into over 90 subtypes), flexibility of the conformational regions, glycan shielding of neutralizing epitopes, the presence of immunodominant non-neutralizing “decoy” epitopes, and the tendency for membrane-solubilized E1E2 antigen preparations to form aggregates [21,24,25,26,27,28,29]. Moreover, direct cell-to-cell transmission of the virus, systemic circulation of virions associate with lipoproteins, and the downregulation of major histocompatibility complex (MHC) expression are other mechanisms for the virus to escape protective immunity [26,30,31,32]. Although immune correlates of protection have yet to be defined for HCV, there is broad agreement that both B and T cell immunity contribute to the control of acute HCV infection [22,33,34]. Thus, the expectation is that an ideal vaccine will elicit high bnAb titers directed against multiple conserved E1E2 epitopes to ensure broad neutralization [35,36,37,38,39], in conjunction with cytotoxic and tissue-resident memory T cells in order to achieve immunity and protection against a high diversity of HCV isolates.

For this review, we will focus on structure-guided approaches to enhance a B cell immune response to E1E2 antigenic determinants that are a primary component of the host defense against HCV infection. It is worth highlighting that such approaches are more complex than sterilizing immunity as observed for hepatitis A, B, and E vaccines [24]. During an acute infection, spontaneous viral clearance occurs in about 25 percent of individuals, which is typically correlated with a robust neutralizing antibody response early in infection [40,41,42,43]. The rate of clearance of a reinfection is improved with a shorter course of infection and an increased likelihood of viral clearance compared with primary infection, suggesting that pre-existing immunity is important [44,45,46,47,48]. In support of this observation, bnAbs passively administered to humanized mice or chimpanzees protects against HCV infection [38,49,50]. Also, passive immunization with anti-HCV antibodies before HCV challenge prevented infection in animal models [38,50,51,52,53]. However, the passive immunization of chimpanzees with antibodies from a HCV-infected patient that neutralized the infectivity of several HCV genotypes in the cell culture only suppressed infection with a homologous virus challenge and failed to protect against heterologous virus strains [51]. Moreover, in a human clinical study using membrane-extracted E1E2 formulated with MF59, an oil-in-water adjuvant, broadly neutralizing antibodies were observed but only in a small fraction of patients in which most patients had low titers and limited the breadth of neutralization [54,55]. Therefore, approaches to enhance immunogenicity such as the development of adjuvant systems that enhance both cellular and humoral immunity and the design of nanoparticle platforms that permit the multivalent presentation of E1E2 and use of novel adjuvants will be essential to achieve immunity against the broad diversity of HCV isolates.

## 2. HCV E1E2 Diversity

**Figure 1:**
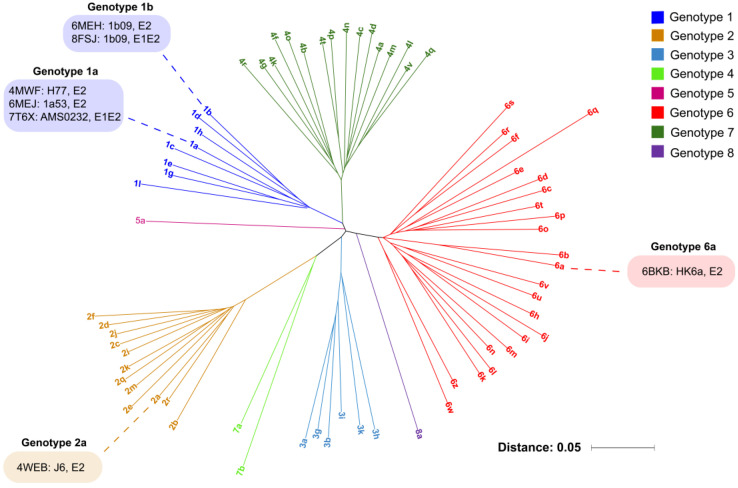
HCV sequence diversity and representative available structures. The HCV E1E2 genotype and subtype reference sequences were downloaded from the LANL HCV database [61] and are shown as an unrooted phylogenetic tree, with branches and labels colored according to the genotype. The subtypes with currently available experimentally determined E2 or E1E2 glycoprotein structures are labeled, with representative structures (Protein Data Bank code, HCV strain, and protein) given. Multiple-sequence alignment and phylogenetic clustering was performed with the MAFFT web server [62], and the unrooted tree was generated with iTOL [63]. Genotype 7b and 8a reference sequences were not available in the LANL HCV reference sequence set and were downloaded from NCBI (Genbank IDs KX092342, MH590698).

As previously noted in the literature [20,31], HCV E1E2 glycoproteins possess high sequence diversity, which is a major reason that HCV is a challenging vaccine target. This diversity is highlighted in Figure 1, which shows a phylogenetic tree generated with representative E1E2 sequences for 69 HCV genotypes and subtypes. As can be seen in the figure, between subtypes of the same genotype, there is often 10% or more in sequence divergence, while greater divergence is observed between genotypes (20–30%). E1E2 sequence variability is not uniform across glycoprotein residues and domains [20,31]; the sequences are punctuated by highly variable regions, including hypervariable regions (HVRs) 1 and 2 and the inter-genotypic variable region (IgVR) in E2. However, other E1E2 regions and sites are highly conserved [20,31], including cysteine residues that form known or putative disulfide bonds, and the majority of N-glycosylation sites (4 sites in E1 and 11 sites in E2 in the genotype 1a H77 strain). Many conserved sites have been confirmed to be important for E1E2 folding and/or function through systematic mutagenesis studies of E2 and E1E2 [56,57,58]. As shown in Figure 1, currently available experimentally determined E1E2 and E2 glycoprotein structures, which are discussed in greater detail in the next section, only account for a few genotypes and subtypes, while they do represent three out of four globally prevalent subtypes noted by others (1a, 1b, 2a, and 3a) [59]. Of note, a recently described but currently unreleased cryo-EM structure contains a modified genotype 3a (S52 isolate) E1E2 [60].

A key feature of HCV diversity is the virus’s capability to form quasispecies in infected individuals and to actively escape the immune response [64]. This immune escape was highlighted in a clinical trial of a neutralizing monoclonal antibody, HCV1, that targets the highly conserved AS412 site (residues 412–423) in E2 [65,66]. Following liver transplantation and monoclonal antibody therapy, which led to dramatic viral reduction, viral rebound was observed in all treated patients along with rare mutations directly within the epitope, at residues 415 or 417 [66,67]. These mutations disrupted HCV1 antibody binding through the mutation of a key epitope side chain or shift in a glycosylation site within the epitope [67]; the latter escape mechanism was also observed for another antibody targeting that E2 site [68]. Other E1E2 polymorphisms associated with resistance and escape have been noted previously [31], with certain polymorphisms outside known epitope sites leading to broad viral resistance or sensitivity [69,70], possibly in some cases due to effects on E1E2 dynamics and putative “open” and “closed” conformational states [71]. While not yet delineated by experimental structural studies, the open and closed states have been associated with the dynamics and conformations of HVR1 on E2, with the more neutralization-sensitive open state becoming prevalent when HVR1 is engaged by the SR-BI coreceptor or deleted by mutagenesis [72].

Recent observations have highlighted that HCV phenotypic diversity, based on the analysis of viral neutralization sensitivity and resistance, often does not directly map onto the overall E1E2 sequence identities and known genotypes and subtypes [73,74,75], likely due in part to the importance of the polymorphisms noted above. These studies have separately identified the representative reference panels of HCV strains, spanning levels of sensitivity and resistance that can prospectively be used to perform standardized assessments of antibodies and immune sera, analogous to a commonly used global panel of HIV strains [76]; however, a single coordinated panel of HCV strains, versus multiple panels, would be advantageous for the HCV research community.

## 3. HCV E2 and E1E2 Structure

**Figure 2:**
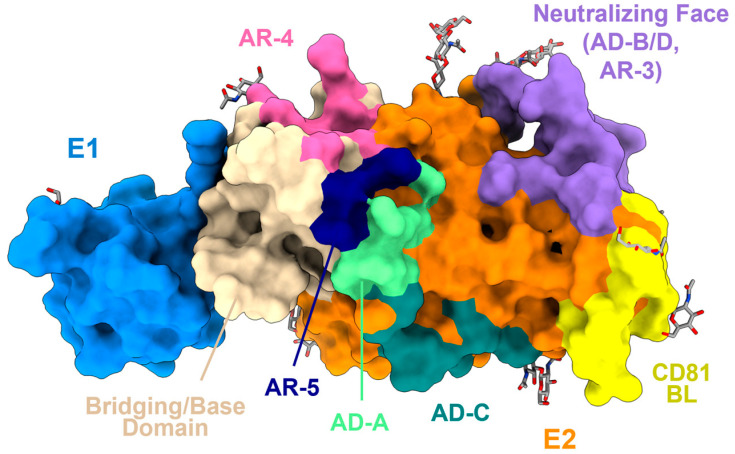
Structure and antigenic regions/domains of the E1E2 heterodimer. Shown is the structure of engineered E1E2 heterodimer ectodomain1, shown in cartoon and semi-transparent surface representation. The E1 and E2 subunits are colored blue and orange, respectively, with mapped antigenic surfaces colored according to the antigenic region (AR) or antigenic domain (AD) targeted: neutralizing face (purple), AR-4 (pink), AR-5 (dark blue), AD-C (teal), and AD-A (light green). The CD81 binding loop and the bridging/base domains are colored yellow and wheat, respectively. N-linked glycans are depicted as gray sticks.

Antibody responses against the HCV E1E2 glycoprotein have been mapped structurally and by mutagenesis to various antigenic domains and regions on the E1 and E2 subunits, which include the targets of broadly neutralizing, strain-specific, and non-neutralizing antibodies [77]. Antigenic targets on the E2 subunit have been named using different nomenclatures, including antigenic domains A–E, antigenic regions 1–5, or epitopes I–III (Figure 2) [38,78]. The most characterized broadly neutralizing antibodies inhibit entry by restricting host CD81 receptor binding and map to a region known as the neutralizing face of E2, which includes overlapping antigenic domains B, D, and E and antigenic region 3 (Figure 2). A contiguous helical region within the stem of E1 also has been defined as a neutralizing antibody target [79,80,81,82]. Antigenic regions 4 and 5, defined by neutralizing antibodies AR4A and AR5A, also confer broad recognition across HCV genotypes but, in contrast to other sites, depend on an intact E1E2 heterodimer for antibody recognition [83].

To date, numerous structures of truncated or modified forms of the E2 subunit ectodomain in complex with antibodies have been determined, including those belonging to genotypes 1a, 1b, 2a, and 6b [9,10,11,12,84,85,86]. The first of these structures, which utilized a truncated E2 protein spanning residues 412–645, defined the overall architecture of the globular E2 core, which was found to contain a central β-sandwich flanked by front and back layers made of loops and short stretches of secondary structure elements [9]. While the core structure of E2 has been found to be largely conserved across genotypes despite sequence diversity, some regions of E2 have been observed to adopt conformational differences, even within the same genotype. These include β-hairpin and extended conformations of the AS412 (domain E) epitope and conformational flexibility of the E2 front layer [13,79,80,86]. Moreover, the CD81 binding site of E2 (residues 418–422 and 520–539) undergoes substantial conformational changes when bound to the large extracellular loop of CD81 [87]. These findings coupled with previous studies showing substantial flexibility and functional interplay between the E2 HVR1, the front layer, and the CD81 binding site, underscore the inherent structural plasticity of some regions of E2 that are thought to underlie differences in susceptibility to antibody neutralization [71,88,89].

**Figure 3:**
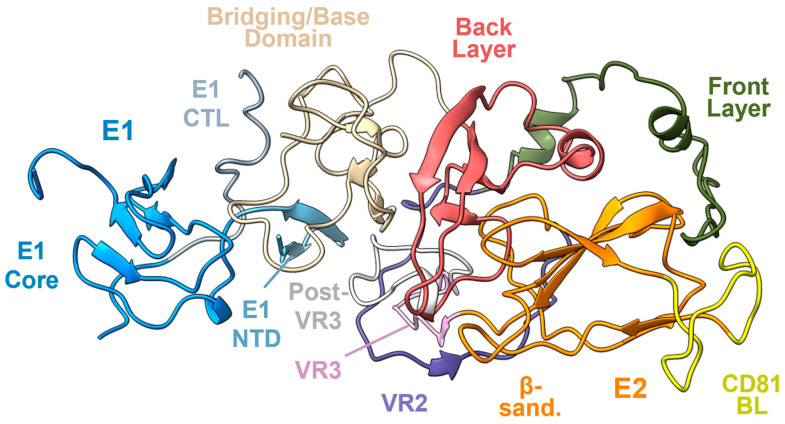
Sub-regions of E1 and E2. Structure of the E1E2 heterodimer shown in cartoon representation and colored according to sub-region (based on PDB ID 8FSJ). The dShown E2 sub-regions include the following: front layer (residues 420–458), variable region 2 (VR2, residues 459–483), β-sandwich core (residues 484–517 and 535–568), CD81 binding loop (CD81 BL, residues 518–534), variable region 3 (VR3, residues 569–579), post-variable 3 region (pVR3, residues 580–595), back layer (BL, residues 596–645), and the bridging (or base) domain (BD, residues 646–701). Shown E1 sub-regions include the following: N-terminal domain (NTD, residues 192–205), E1 core (residues 206–255 and 295–299), and C-terminal loop (CTL, residues 300–314).

In order to structurally characterize the HCV E1E2 envelope complex in a more native-like form, cryo-EM has recently become the technique of choice (Figure 3). Notably, this technique yielded the first structure of a full-length membrane-extracted form of the E1E2 heterodimer of genotype 1a bound by neutralizing antibodies [18]. This structure resolved the overall configuration of the E1E2 heterodimer, including regions of E1 and the C-terminus of E2, and defined, for the first time, the AR4 site of vulnerability. Subsequently, our group determined a cryo-EM structure of sE1E2.SZ (of genotype 1b), an engineered soluble form of the E1E2 heterodimer ectodomain that we developed for vaccine efforts by replacing the transmembrane domains of E1 and E2 with a soluble self-assembling coiled-coil scaffold (Figure 3) [19]. Both heterodimer structures were found to share a similar E1E2 fold and architecture despite belonging to different genotypes and diverging in amino acid sequence by ~20%. The liberation of sE1E2.SZ from membrane therefore preserved its native-like conformational state [19]. Analysis of the structures revealed several common features of the E1E2 heterodimer. The E1–E2 interface was made up entirely of non-covalent, predominantly hydrophobic interactions. A substantial contribution of two conserved E1 N-linked glycans, N196 and N305, to the interface accounted for roughly a third of the E1 interface with E2 [19]. In both structures, a portion of the C-terminal domain of E2 was resolved (residues 646–704), termed the base or bridging domain, and was found to pack against the back layer of the E2 core and against E1, accounting for roughly 70% of the E2 interface with E1. This domain also contained the epitope of E1E2-specific neutralizing antibody AR4A, which was found to be one of the most highly conserved epitopes defined to date [19].

Several domains of E1 were also resolved in the E1E2 structures [18,19]. The E1 N-terminal domain (NTD), spanning residues 192–205, was found to consist of two anti-parallel β-strands that packed against variable region 2, post-variable region 3, and the bridging/base domain of E2. The E1 NTD also contained the N-linked glycan at position N196, which, along with N305, was previously found to be critical for heterodimer integrity [58,81,90]. The E1 core domain, residues 206–255, consisted of a cluster of β-strands and contained two N-linked glycans, N209 and N250. The C-terminal loop (CTL) domain of E1, spanning residues 295–312, contained the N305 glycan that, as noted above, packed against the bridging/base domain of E2. The CTL also contained the epitope for E1-specific neutralizing antibodies IGH520/IGH505 and IGH526 [18,79,80,81,82]. Missing from both structures, however, was the region linking the E1 core and C-terminal loop (residues 256–294), which has been proposed to contain the putative fusogenic machinery [91,92,93].

Recently, a cryo-EM structure of a full-length membrane-extracted dimer of E1E2 heterodimers has also been reported, although not yet released, indicating that possible higher-order oligomers may represent the native state of the E1E2 glycoprotein on HCV virions [60]. Interestingly, flexible regions of E2 and E1 that were not resolved in previous structures, including parts of HVR1, AS412, and membrane-embedded portions of the E2 and E1 stem regions, were reportedly resolved in this structure. The structural definition of these regions will likely provide further insight into the underlying structural features of E1E2 that play a role in membrane fusion and possibly those that define phenotypic differences in susceptibility to antibody neutralization [60].

## 4. Structure-Based Vaccine Design

**Figure 4:**
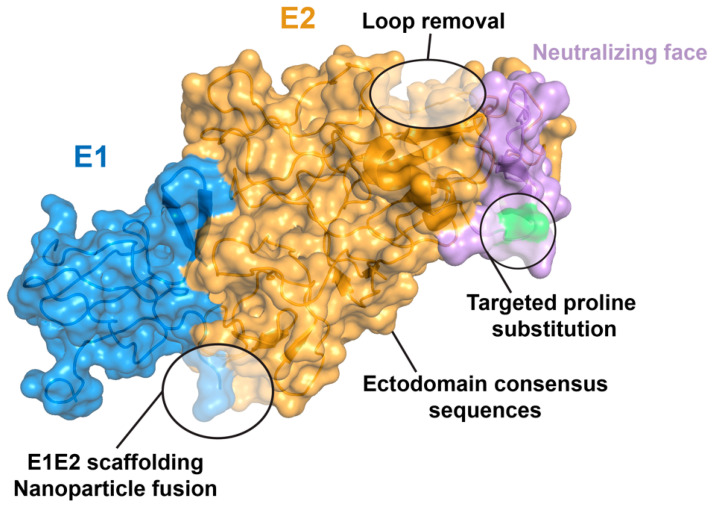
E2 and E1E2 structure-based and rational design strategies. Selected reported E2 and E1E2 design approaches are shown on an E1E2 structure (PDB code 8FSJ), with E1, E2, and neutralizing face (antigenic domain B/D, antigenic region 3) colored as in Figure 2. E2 position 445, the site of a previously reported neutralizing face proline design [106], is colored green. E1E2 scaffolding and nanoparticle fusion are shown at the E1 and E2 C-termini, while loop removal is shown at the E2 HVR2 loop.

To generate an effective vaccine for HCV and overcome the challenges of HCV diversity and immune evasion [31], recent efforts have increasingly explored the use of reverse vaccinology and structure-based vaccine design [94,95] to generate optimized vaccine antigens that will elicit broadly neutralizing antibodies that target key conserved sites on E1E2 [96]. These follow successful structure-based designs of antigens for other variable or dynamic viruses, including prefusion RSV F [97] and influenza hemagglutinin stem [98] antigens, which have both been in clinical trials, and recently approved for use in the case of prefusion RSV F [99,100]. Structure-based HCV antigen designs have included stabilized and scaffolded conserved epitopes [101,102,103], optimized E2 antigens with truncated or removed variable regions [104,105] or a targeted proline substitution to stabilize a key epitope [106], as well as display of E2 self-assembling protein nanoparticles [104]; these and other HCV antigen designs and strategies are described in a review by Guest and Pierce [96]. One recent success in this area is the design of stabilized and secreted E1E2 ectodomains with a coiled-coil scaffold, which maintained the antigenicity of native full-length E1E2 and elicited neutralizing antibodies [107,108]. Others designed a soluble “E2E1” construct with E1 and E2 ectodomains permuted, which was presented on self-assembling nanoparticles in a mosaic format representing six HCV strains; while this successfully elicited cross-neutralizing antibodies in vivo, the lack of binding by E1E2-specific antibody AR4A indicates that the permuted design does not reflect native E1E2 assembly [109]. Some E2 and E1E2 design strategies are shown in Figure 4.

Several recent advances and findings provide possible avenues to pursue in future HCV antigen design efforts. Importantly, the current availability of experimentally determined E1E2 structures, as discussed in the previous section, enables the structure-based design of E1E2, rather than E2 alone or individual epitopes as in previous work, to optimize its stability, antigenicity, or other features. Additionally, putative “open” and “closed” states of E2 and E1E2 [71], or HVR1 entropy [88], which, as noted above, have been associated with viral neutralization sensitivity, can be utilized to tune E1E2 antigenicity, particularly if sufficient structural and dynamic details underlying those states can be defined. Of relevance, a recently described cryo-EM structure with E1E2 in dimeric form seems to provide details of a preferred conformation of HVR1 which was corroborated by AlphaFold2 structural modeling [60].

Other (non-structural) rational antigen design approaches represent promising strategies to address HCV diversity and escape. Frumento et al. identified E1E2 ectodomains associated with spontaneous viral clearance and improved neutralizing antibody breadth [110] that may useful in a vaccine, versus the H77 glycoprotein sequences which are commonly used in E2 and E1E2 antigens. Another strategy is the use of consensus ectodomains, which was utilized in HCV E2 [111] and HIV envelope glycoprotein [112,113] antigen designs. Finally, an additional means to address HCV diversity could be the use of multiple representative designed or natural E1E2 antigens in a vaccine that are representative of prevalent genotypes; given the previous success of self-assembling mosaic nanoparticles to display diverse coronavirus spike receptor binding domain antigens [114], an analogous strategy could be explored for displaying diverse representative HCV antigens, as recently reported [109]. Due to the wide diversity of HCV E1E2 sequences between and within genotypes, it is not clear whether a single E1E2 sequence (consensus or naturally derived) would effectively represent the breadth of HCV genotypes or subtypes in the context of a vaccine, versus targeting individual genotypes or subtypes, or utilizing a set of representative immunogens. However, given the many known broadly neutralizing monoclonal antibodies that target conserved E1E2 sites [38,56,83,115,116,117] that would be present on most E1E2 antigens, it is likely that single antigens can elicit such broadly neutralizing antibodies, as seen in mice, non-human primates, and humans [54,108,118]. Whether the antibodies induced by such antigens can provide sufficiently effective and broad protection is an open question; future studies investigating and comparing the immunogenicity and protection of these strategies and approaches can help address this.

## 5. Multivalent Delivery Platforms and Considerations

Subunit vaccines are often poorly immunogenic; a phenomenon routinely attributed to their size as particulate antigens have been known for quite some time to be highly immunogenic [119,120,121,122,123]. There is recent data in a study by Aung et al. [124] that puts a finer point on this concept. In that study, the authors showed that subunit vaccines were trafficked primarily to the subcapsular sinus or extracellular regions of lymph nodes and subsequently degraded by metalloproteases. Such degradation eliminates conformation-dependent epitopes on the associated antigens and hampers the immune response. Nanoparticle-sized antigens were localized instead to follicular dendritic cells (FDCs) where they remained intact and preserved such conformation-dependent epitopes and thus elicited a more robust immune response. In light of these recent observations and the historical data, it seems clear that increasing the size of a subunit vaccine is beneficial and thus a number of strategies have been developed toward that end. One strategy is to employ an adjuvant system that, when formulated with the antigen, produces a nano- or microparticulate vaccine. Common adjuvant systems in use to make particulate subunit vaccines are aluminum salts (Alum), polymers like poly(D,L-lactic-co-glycolic) acid (PLG), oil and water emulsions like MF59, liposomes and other vesicles, and micelle-forming adjuvants. Micelles can be particularly advantageous for membrane-anchored antigens as the formulation process creates rosettes of antigens covering the exterior of the micelle. These kinds of formulations have been used in vaccines for influenza (Flublok) and SARS-CoV-2 (NVX-CoV2373) [125,126,127,128,129,130,131]. A second strategy to develop particulate vaccines is to construct virus-like particles (VLPs). VLPs are non-infectious but more closely mimic the native virion than subunit vaccines and other particulate platforms by using viral structural proteins to self-assemble in a similar manner to the virus. Energix (hepatitis B virus) and Gardasil (human papilloma virus) are two prominent VLP-based vaccines. A third strategy for increasing the size of a subunit vaccine is the use of protein-based nanoparticles. These nanoparticle platforms are typically naturally occurring or engineered protein shells that allow for the multivalent display of subunit vaccines on the exterior [132,133,134,135,136,137,138]. These assemblies can be formed in cis, where the subunit vaccine and nanoparticle protomer are expressed as a single open reading frame and assembly yields a 100% occupied nanoparticle, or in trans where the nanoparticle shell and subunit vaccine are produced separately and coupled post hoc, as in the case of the SpyCatcher-SpyTag system [139]. Each of these platforms is being explored for a potential HCV vaccine candidate [104,109,140]. The first studies by Yan et al. [140] and He et al. [104] used the E2 ectodomain and a modified E2 core ectodomain, respectively, as proof-of-principle antigens to be appended to nanoparticles. Given the importance of the E1E2 complex, and in particular the AR4/AR5 antigenic region in viral clearance [37,110], a nanoparticle-presenting native E1E2 should be a high priority for HCV vaccine development. One nanoparticle study has been conducted with E1 and E2 [109], but this used a permuted E2-E1 version of the antigen which does not retain the native AR4/AR5 antigenic domain.

**Figure 5:**
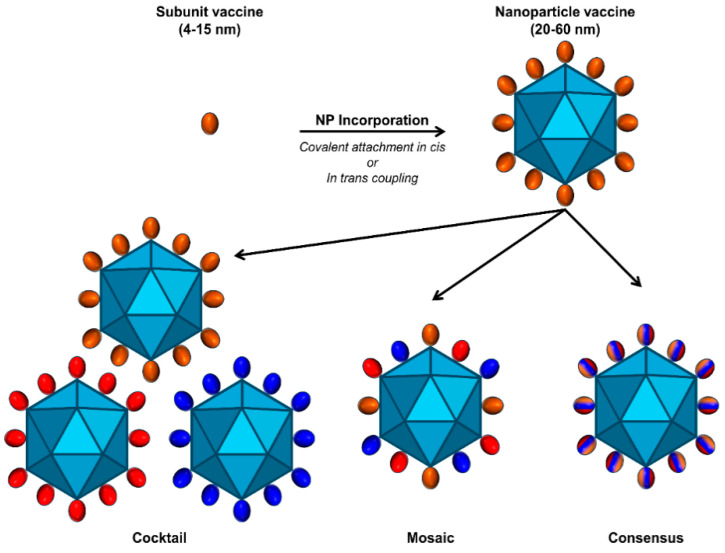
Multivalent presentation of E1E2 antigens. Coupling to a nanoparticle increases the size and valency of E1E2 antigen presentation. Flexible attachment methods allow for the presentation of genetically diverse sequences as consensus (striped), mosaic (multiple colors), or cocktails of multiple representative genome sequences.

While the above platforms address the question of size in the context of a vaccine against HCV, the question that still remains is how to accommodate HCV diversity in the context of a particulate adjuvant, a VLP, or a nanoparticle. As described in the previous section, this problem can be overcome by the use of consensus sequences or mosaic vaccines composed of multiple antigens encompassing different genotypes or phenotypes contained within a single vaccine (Figure 5). These approaches have been used for vaccine trials against HIV and SARS-CoV-2 [114,141,142,143,144,145,146,147,148,149] and, importantly, are compatible with the adjuvant and nanoparticle platforms, but it is unclear if such approaches are compatible with VLPs. An additional approach is to use cocktails of different genotypic or phenotypic representatives mixed together after preparation and validation. This is the most straightforward approach but requires a brute-force regimen of multiple separate preparations and validations for each different member of the cocktail. Which one of the above approaches is most likely to yield an effective HCV vaccine is currently an open question that will need to be evaluated experimentally.

## 6. Conclusions

Since its discovery in 1989, HCV has been a particularly vexing pathogen for vaccine development, in large part due to its high sequence diversity. However, significant advances have provided avenues to potentially overcome this. First, despite significant sequence variability among the different genotypes and subtypes, highly-conserved regions have been defined and characterized as antigenic regions [38,83,150,151,152,153,154,155,156,157,158] that give rise to bnAbs and can serve as potential targets for rational vaccine design. Second, after significant struggles, structural studies were successful first for modified truncated versions of the E2 ectodomain [9,10], and subsequently for a more complete E2 ectodomain [11] in complex with neutralizing and non-neutralizing antibodies. More recently, the structure of membrane-extracted E1E2 in a complex with multiple bnAbs was determined by cryo-EM [18], providing the first look at the antigenic domain AR4, which is bound by the bnAb AR4A, and correlates with viral clearance [37,110]. In addition, our group developed a soluble, secreted form of the E1E2 complex, with the idea that an E1E2 antigen liberated from the membrane would prove more amenable to vaccine design efforts. The structure of the secreted E1E2 complex [19] shows that it preserves the native architecture of the E1E2 ectodomain outside the context of the membrane. This catalogue of structures (plus other structures likely to be determined) can be used for structure-based vaccine design of an E1E2-based HCV vaccine. Third, the development of nanoparticle platforms, both protein- and adjuvant-based, allows a multivalent presentation of E1E2 as a means to enhance its immunogenicity. Moreover, advances in these nanoparticle platforms such as plug-and-display technology [139] allows for the potential development of mosaic E1E2 vaccines. It is not known what means of incorporating sequence diversity into an E1E2 vaccine will be successful, so making as many options as possible available is critical for successful vaccine development. Additional improvements such as incorporating pan DR epitope (PADRE) peptides [159,160,161,162] into nanoparticles could potentially boost the cellular immune response to an E1E2-based vaccine, thereby further enhancing its potency. PADRE is an artificial T-cell epitope that elicits a strong CD4+ T helper response in vitro, and can therefore be used to boost the overall cellular immune response without perturbing the nanoparticle system. Like pieces of a puzzle, these and other breakthroughs from research on HCV and other pathogens have come together to put the field of HCV vaccine development in a position to overcome the hurdle of HCV sequence diversity. This progress has the potential to deliver a HCV vaccine that elicits the breadth of neutralization required to achieve containment and eventual eradication.

## Data Availability

Not applicable.
